# 

**Published:** 1880-01

**Authors:** 


*CONTENTS.*

| | Page |
|---|---|
| Pneumonia | 1 |
| Care of Children | 3 |
| Health Capital | 3 |
| Facts Worth Remembering | 4 |
| A Recent Cough | 5 |
| To Stop Bleeding | 5 |
| A Severe Cold | 5 |
| A Lay Sermon | 6 |
| Scientific Notes | 8 |
| An Anti-Fat | 8 |
| Feeding Horses | 9 |
| Pure Water | 11 |
| How to Get Pure Water | 12 |
| Putting Coins in the Mouth | 13 |
| Book Notices | 14 |
| Eating and Drinking | 15 |
| Consumption | 15 |
| Ventilation | 22 |
| How to Argue | 22 |
| Vermin Riddance | 23 |
| Intussusception | 23 |
| Human Health | 24 |
| Medication and Nutrition by Absorption | 25 |

